# Hématome rétro-péritonéal consécutif à une envenimation vipérine: à propos d’un cas

**DOI:** 10.11604/pamj.2019.32.70.17951

**Published:** 2019-02-08

**Authors:** Boubacar Diallo, Mohamed Keita, Hammadoun Dicko, Mamadou Karim Toure, Seydina Alioune Beye, Joseph Kone, Daouda Diallo, Abdoul Hamidou Almeimoune, Moustapha Issa Mangane, Sory Traore, Abdoulaye Traore, Birama Togola, Diénéba Doumbia, Youssouf Coulibaly

**Affiliations:** 1 Service d'Anesthésie-Réanimation, CHU Point G, Bamako, Mali; 2 Département d'Anesthésie-Réanimation et Urgences, CHUME Luxembourg; 3 Service d'Anesthésie-Réanimation, CHU de Kati, Mali; 4 Département d'Anesthésie-Réanimation et Urgences, CHU Gabriel Toure, Bamako, Mali; 5 Service de Chirurgie B, CHU Point G, Bamako, Mali

**Keywords:** Envenimation vipérine, hématome rétro-péritonéal, réanimation, Viperine envenomation, retroperitoneal haematoma, resuscitation

## Abstract

Les envenimations ophidiennes constituent un problème de santé publique en Afrique, entrainant 20 000 décès annuels. Cette mortalité traduit les difficultés de prise en charge des complications en particuliers hémorragiques. Nous rapportons le cas de Mr SL 35 ans, agriculteur, victime d'une morsure de vipère entrainant un syndrome hémorragique, suivi d'un abdomen aigu d'installation progressive. L'échographie objectivait un épanchement de grande abondance, dont la ponction ramenait du sang incoagulable. Devant la reconstitution de l'hémopéritoine après trois doses de sérum antivenimeux et la coagulation des prélèvements sur tube sec, une laparotomie exploratrice était réalisée à J9. Elle a retrouvé un hématome retro péritonéal bombant dans le méso-colon et qui s'écoulait dans la cavité péritonéale. Le geste a consisté à une évacuation de 1500ml de sang, et un toilettage au sérum physiologique. Les suites opératoires ont été simples. Le patient était transféré en secteur d'hospitalisation de chirurgie à J3 post opératoire puis sortie d'hôpital à J19.

## Abstract (en)

Ophidian envenomations are a public health problem in Africa, causing 20.000 annual deaths. This mortality reflects the difficulties in the management of these complications, in particular of bleeding complications. We report the case of Mr SL, aged 35 years, farmer, victim of snake bite causing haemorrhagic syndrome followed by a progressive onset of acute abdomen. Ultrasound objectified high abundant effusion. Effusion puncture was performed which showed unclotting blood. Given the re-establishment of the hemoperitoneum after three doses of antivenin serum as well as the coagulation of the samples collected using drying tube, exploratory laparotomy was performed on day 9. It showed retroperitoneal hematoma, with bulging appearance, in the mesocolon which flowed in the peritoneal cavity. Aspiration of 1500ml of blood and rinsing with physiological serum were performed. The postoperative course was uneventful. The patient was transferred to the department of surgery three days after surgery and was discharged on day 19.

## Introduction

Les morsures de serpent constituent une urgence médico-chirurgicale fréquente et un véritable problème de santé publique. Pour une population de 750 millions de personnes, un million de morsures de serpent surviennent chaque année en Afrique entraînant 600 000 envenimations. Plus de 20 000 décès sont enregistrés [1]. L'hémorragie peut émailler l'évolution [2]. Dans la littérature les signes d'hémorragie extériorisée sont plus décrits [3]. Mensah et al., au Benin rapportent en 2004, un cas d'hémopéritoine consécutive à une morsure vipérine [4]. Dans nos pays, la prise en charge se heurte à une insuffisance du plateau technique, de ressources humaines et le manque de moyens de la population. Nous rapportons un cas d'hématome retro-péritonéal constitué 5 jours après une envenimation par morsure de vipéridae.

## Patient et observation

M. SL âgé de 35 ans, sans antécédent pathologique connu, agriculteur habite à 200Km de Bamako. Il a été admis en réanimation pour hémopéritoine. Dix jours avant son admission il avait été victime d'une morsure de vipère à l'auriculaire gauche lors de travaux champêtres. Par la suite s'est installé progressivement un syndrome hémorragique fait de gingivorragie, d'hématurie, un saignement local et un œdème extensif dépassant l'épaule et atteignant l'hémi thorax. L'évolution était favorable après une dose de sérum antivenimeux (SAV). Au cinquième jour il est admis au centre de santé du district pour une distension abdominale sans notion de traumatisme. Le bilan fait en urgence objectiva une hémoglobinémie à 8,7g/dl et un épanchement intra-péritonéal de moyenne abondance à l'échographie abdominopelvienne. Devant ce tableau une dose de sérum antivenimeux était administrée. L'évolution était marquée par la persistance de la douleur et la distension abdominale d'aggravation progressive motivant son transfert en réanimation du CHU du Point G. A l'évaluation clinique le patient était anxieux, un syndrome anémique fait de pâleur conjonctivale, une tachypnée à 26c/mn, une tachycardie à 113bts/mn, une distension abdominale avec une sensibilité à la palpation. Il était stable sur le plan hémodynamique. Les urines étaient claires au sondage urinaire. Le prélèvement de sang sur tube sec était incoagulable. A la biologie on notait une anémie normocytaire normochrome à 3,8g/dl; une leucocytose à 16700/mm^3^, les plaquettes à 326000/mm^3^. La créatininémie était à 174μmol/l et l'urée sanguine à 20,90mmol/l.

**Figure 1:**
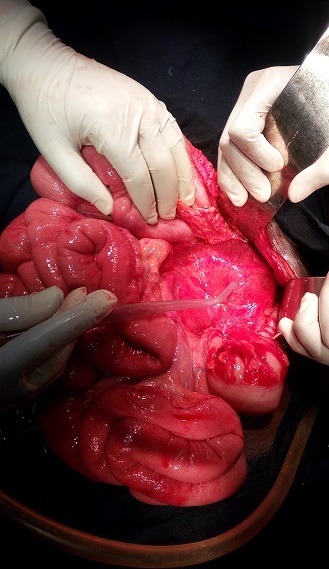
bombement du rétro-péritoine par l'hématome

**Figure 2:**
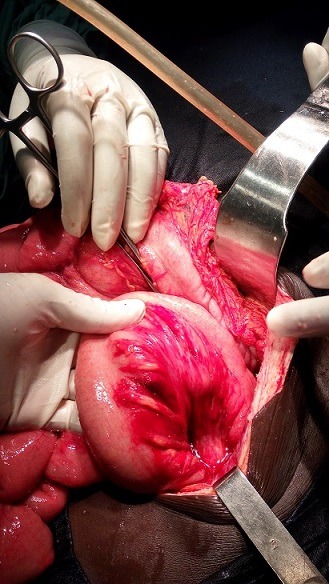
infarcissement mésentérique diffus sans saignement actif

La prise en charge a consisté à une sérothérapie, une transfusion de 3 culots globulaires et de 2 plasmas frais congelés et une antibiothérapie par amoxicilline, et la réhydratation. Devant la persistance de la distension abdominale, après trois doses de SAV et la coagulation des prélèvements sur tube sec, une échographie de contrôle fut réalisée et objectivait un épanchement de grande abondance qui se reconstituait après chaque drainage. Le taux de prothrombine et le temps de céphaline plus activateur, réalisé tardivement pour des raisons techniques, étaient respectivement de 61%, et 27,9 sec. Une laparotomie exploratrice était réalisée à J9 sous anesthésie générale. Elle a retrouvé un hématome retro péritonéal bombant dans le méso-colon (Figure 1) de part et d'autre qui s'écoulait dans la cavité péritonéale, et un infarcissement mésentérique diffus (Figure 2) sans saignement actif. Le geste a consisté à une évacuation de 1500 ml de sang, un toilettage au sérum physiologique et la mise en place de deux drains dans le rétro péritoine. Les suites opératoires ont été simples. Le patient était transféré en secteur d'hospitalisation de chirurgie à J3 post opératoire puis sortie d'hôpital à J19 post opératoire.

## Discussion

Les vipéridés sont responsables de plus de 90% des envenimations ophidiennes en Afrique au sud du Sahara [5]. Parmi les espèces retrouvées on note une prédominance des Bitis et des Echis. Le venin de vipères est un mélange complexe d'enzymes protéiques et de toxines en quantités variables [6]. Il peut être responsable d'atteinte clinique et/ou biologique de l'hémostase, et d'un syndrome local (douleur, œdème, nécrose et/ou gangrène). Ces manifestations constituent une entité syndromique appelée «syndrome vipérin» [7]. Les venins d'*Echis* et de *Bitis* sont riches en protéines agissant sur l'hémostase avec notamment de nombreuses enzymes [8]. L'inhibition des plaquettes réduit leur efficacité tandis que l'activation pathologique réduit le nombre de plaquettes circulantes, d'où une thrombopénie [9]. Les protéines interférant avec la coagulation sont distinguées entre protéases pro-coagulantes et protéases anticoagulantes. Les protéases pro-coagulantes se substituent aux facteurs de coagulations ayant des propriétés analogues. L'activation du processus de coagulation qui en découle, persiste jusqu'à l'épuisement d'un ou plusieurs facteurs aboutissant à un syndrome hémorragique par afibrinogénémie [10]. Aussi il existe des protéines pouvant activer la fibrinolyse, les enzymes fibrinolytiques. Elles possèdent des propriétés similaires à la plasmine et sont susceptibles d'hydrolyser à la fois le fibrinogène et la fibrine [10, 11].

Les manifestations liées à ces différentes perturbations de l'hémostase peuvent être simples (saignement local, gingivorragie…) à sévère (hémorragie persistante voire état de choc) avec ou sans hématome profond (cérébral, péritonéal, voire retro péritonéal) comme rapporté dans notre cas. En général l'hémorragie extériorisée est la règle avec une gravité influencée par plusieurs facteurs [12]. La toxicité du venin et la quantité inoculée par le serpent en sont les éléments essentiels. Le délai de prise en charge a également de grandes conséquences. Tout retard est source de complications et réduit l'efficacité du traitement dans des proportions difficiles à évaluer [13]. L'installation du syndrome hémorragique est plus souvent insidieuse. Il débute par un écoulement sanguin discret et permanent. Le défaut de coagulation se traduit par un purpura ou par des hémorragies qui peuvent être extériorisées, cérébrales, voire viscérales profondes [1]. L'évolution vers une anémie sévère ou un choc hypovolémique peut engager le pronostic vital.

Le cas que nous rapportons semble exceptionnel de par la localisation de l'hématome, le délai long de constitution (5 jours) et en dépit de la sérothérapie initiale. Sa gravité était moindre comparée au cas d'hémopéritoine rapporté par Mensah E *et al.* [4] admis en choc hémorragique 12 heures après la morsure. Ces différences pourraient s'expliquer par la sérothérapie initiale précoce dans notre cas. La réalisation d'un geste chirurgical chez un patient présentant un syndrome hémorragique par envenimation reste une situation délicate. Elle ne s'envisage qu'après une correction des troubles de l'hémostase par l'immunothérapie antivenimeuse [12]. L'apport de facteur de coagulation peut être envisagé selon la biologie. La poursuite de l'immunothérapie est indiquée devant la persistance de l'hémorragie, une fibrinogénémie inférieure à 1g/L, un TP inférieur à 50%, un TCA supérieur à 1,5 fois le temps du témoin [12]. En l'absence de laboratoire, la coagulation obtenue sur un tube sec au lit du malade permet d'effectuer la surveillance du traitement [13]. Dans notre cas, la sérothérapie était renouvelée en fonction du test de coagulation sur tube sec dont la normalisation était obtenue après trois doses. Malgré l'existence de protocole bien codifié, la prise en charge des envenimations ophidiennes dans nos pays, se heurte à beaucoup d'obstacles. L'organisation et l'accessibilité des centres de santé, l'équipement et l'approvisionnement en médicaments restent problématiques et difficiles. A cela s'ajoute le coût élevé des sérums antivenimeux avec une conservation difficile en périphérie compromettant ainsi l'efficacité thérapeutique [14]. Toute chose rendant la prise en charge difficile et périlleuse.

## Conclusion

Les complications d'une envenimation vipérine peuvent être graves avec une symptomatologie variée. Le cas rapporté en est une illustration parfaite qui mérite d'être recherché par les soignants. Une prise en charge rapide et adaptée permettra certainement de réduire sa survenue.

## Conflits d’intérêts

Les auteurs ne déclarent aucun conflit d'intérêts.
